# Antitumor effects of Dasatinib on laryngeal squamous cell carcinoma in vivo and in vitro

**DOI:** 10.1007/s00405-013-2394-3

**Published:** 2013-02-13

**Authors:** Yan Song, Xin Sun, Wei-Liang Bai, Wen-Yue Ji

**Affiliations:** Department of Otolaryngology, Sheng-Jing Hospital, China Medical University, 110004 Shenyang, People Republic China

**Keywords:** Head and neck cancer, Dasatinib, Hep-2 cell line, Apoptosis

## Abstract

A novel drug named Dasatinib is a highly potent ATP-competitive orally active dual Src/Abl kinase inhibitor with anti-proliferative activity against solid tumors and CML (chronic myeloid leukaemia) cell lines. Dasatinib has been shown to have preclinical activity against human prostate, breast, pancreatic, lung, and head and neck cancer. To determine whether Dasatinib can inhibit the growth of laryngeal squamous cell carcinoma, in the present study, we investigated the antitumor effect of Dasatinib on Hep-2 cells. Hep-2 cells were treated with different concentrations of Dasatinib for different time. Cell proliferation, cell cycle distribution, and cell apoptosis were evaluated using MTT assay, flow cytometry, and fluorescent microscopy. It was found that Dasatinib exhibited significant efficacy in growth inhibition, cell cycle arrest at G0/G1 phase, and apoptosis induction in a dose- and time-dependent manner. Measuring the modulation of regulators in the cell cycle, apoptosis and signal transductions by western blot analysis showed that the effect of Dasatinib was due to suppression of the expression of Bax, Bcl-2, Caspase-3, and Caspase-8. Moreover, in vivo studies were performed in a nude mouse xenograft model, the new prescription (DDP + Dasatinib) was better than DDP alone in terms of therapeutic efficacy. In conclusion, the antitumor effect of Dasatinib on Hep-2 cells was due to the induction of cell cycle arrest as well as apoptosis. The possible mechanisms underlying the action might be attributed to the suppression of Src phosphorylation. This investigation suggests a potential clinical application of Dasatinib for the treatment of laryngeal cancer patients

## Introduction

Laryngeal squamous cell carcinoma is one of the most common squamous cell carcinomas of the head and neck. Laryngeal carcinoma ranks the 11th common kind of cancer in men worldwide, with a tendency toward an increasing occurrence of new cases and deaths annually [1]. More than 95 % of all laryngeal cancers are squamous cell carcinomas. The traditional treatment for squamous cell carcinoma of the larynx was surgery, usually total laryngectomy in advanced cases, followed by radiotherapy. During the last decades, combined modality approaches have been developed, and chemotherapy has been confirmed as a major component of these treatment approaches to enhance loco-regional disease control, reduce distant metastasis, and improve survival in patients with advanced laryngeal cancer [2]. Therefore, the therapeutic strategies, which are able to produce greater efficacy and preserve larynx as well, are essential to this concept [3]. However, potential complications and side effects of this drug limit its application in Laryngeal carcinoma chemotherapy, which may account for the failure of chemotherapy in patients with advanced laryngeal carcinoma. In this regard, novel effective chemotherapeutic agents are desperately needed in the treatment of laryngeal cancer to improve survival and to enhance larynx preservation.

Src is the prototypic member of a family of nine non-receptor tyrosine kinases [SFKs (Src family kinases), which include Src, Lyn, Fyn, Lck, Hck, Fgr, Blk, Yrk, and Yes] that has a key role in many cellular signaling pathways [4]. SFKs regulate four main cellular functions that ultimately control the behavior of transformed cells: cell proliferation, adhesion, invasion, and motility [5]. Src is overexpressed in head and neck squamous cell carcinoma relative to control tissues. Src offers particularly a promising molecular target for anticancer therapy, so we want to use Src kinase inhibitor to inhibit the signaling pathways and to decrease the growth of laryngeal carcinoma.

A novel drug named Dasatinib (BMS-354825, SPRYCEL^®^; Bristol-Myers Squibb) is a highly potent ATP-competitive orally active dual Src/Abl kinase inhibitor with anti-proliferative activity against solid tumors and CML (chronic myeloid leukaemia) cell lines. It sensitively inhibits all members of the Src family, including c-Src, Lck, Fyn, and Yes. At higher concentrations (3–28 nmol/L), Dasatinib also inhibits the Src kinases Abl, c-Kit, PDGFR, and EphA2 [6]. Dasatinib has been shown to have preclinical activity against human prostate, breast, pancreatic, lung, head and neck cancer, mesotheliomas, and sarcomas dependent on Src kinase [7].

To date, no study has been conducted in the evaluation of the effect of Dasatinib on human laryngeal cancer cells. As constitutive activation of P-Src has been determined in head and neck squamous cell carcinoma, and blockade of Src signaling has been shown to effectively inhibit cell growth and induce apoptosis in head and neck squamous cell carcinoma cells, we hypothesized that Dasatinib would be useful in treating human laryngeal cancer cells. To test this hypothesis, we designed the present study to examine the antitumor effect of Dasatinib in vitro and in vivo on human laryngeal cancer cells, including the effects on cell growth, cell cycle distribution, apoptosis, and the expression of proteins relevant to the regulation of cell cycle and apoptosis pathway.

## Materials and methods

### Reagents

Dasatinib (Mr, 488.0) was obtained from Bristol-Myers Squibb. For in vitro use, Dasatinib was dissolved in DMSO at a concentration of 10 mg/mL. The solution was aliquoted and kept at −20 °C until use. Frequent freeze-thawing was avoided. For in vivo use, Dasatinib was dissolved in citrate buffer and this solution was kept at 0–4 °C for up to 14 days. Cisplatin (EMD Biosciences, Inc.) was dissolved in 10 mg/ml of DMSO for both in vitro and in vivo use. 3-(4,5-dimethylthiazol-2-yl)-2,5-diphenyl tetrazoliumbromide (MTT) was from Fluka, USA. Propidium iodide (PI) was from Biosharp, USA. Annexin V-FITC apoptosis detection kit was from Jingmei Biotech Co. Ltd., Shenzhen, China. Bcl-2, Bax, caspase-3, caspase-8, and actin primary antibodies, as well as the secondary antibody were obtained from Santa Cruz Biotechnology Inc., CA, USA. Anti-phosphorylated Src antibody (p-Src Tyr 416) and total Src antibody were both obtained from Cell Signaling Technologies. Dulbecco’s modified Eagle’s medium (DMEM), and fetal calf serum (FCS) were obtained from Gibco BRL (Grand Island, NY, USA). Other laboratory reagents were obtained from Sigma, USA. Fluorescence microscopy (Olympus, Japan) and flow cytometry (FACScalibur, Becton–Dickinson, USA) were employed.

### Animals

Twenty male nude mice (3 months old, weighing 20 ± 2 g) were obtained from China Medical University animal facility and fed with purified water and a commercial stock diet in an air-conditioned room at 20–22 °C. All the animals were treated softly and equally. All animals were killed by cervical dislocation method. All the research was approved by The Institutional Animal Care and Use Committee of China Medical University.

### Cell culture

Human laryngeal cancer line Hep-2 was bought from Cell Bank, Shanghai Institutes for Biological Sciences, Chinese Academy of Sciences, Shanghai and cultured in RPMI 1640 supplemented with 10 % FBS, 100 U/ml penicillin G, and 100 μg/ml streptomycin. Cultures were maintained in a 5 % CO_2_ humidified atmosphere a 37 °C.

### Cell growth inhibition assay

The MTT assay was performed to assess viability of the human laryngeal cancer line Hep-2 after treatment with 0.1, 1 and 10 mg/ml Dasatinib. 96-well plate cultures, after exposure to the control or test solutions for a specific time period (observed at 12, 24, and 36 h), were incubated with 50 mg/ml MTT at a dilution of 1:10 based on the volume of culture medium for 3 h at 37 °C. At the end of the incubation time, the MTT solution was removed and 150 ml DMSO was added to each well and stirred to dissolve the dark-blue formazan crystals which had formed. The proportion of viable cells was determined by measuring the optical density of each sample at 480 nm with a spectrophotometer. Three cultures were exposed to each solution at each time period. The means of each group of cultures were compared.

### Cell cycle analysis by flow cytometry

Cell cycle analyses were carried out using DNA flow cytometry. Hep-2 cells (1 × 10^6^/well) were seeded in six-well plates and allowed to attach overnight. Cells were treated with 0.1, 1 and 10 mg/ml Dasatinib for 24 h and harvested by 0.25 % trypsin. The cells were then washed twice with PBS, centrifuged at 1,000 *r*/min for 5 min, and fixed in 70 % ethanol at 4 °C. Before DNA analysis, the cells were washed again with PBS, treated with 50 μg/ml of RNase, and stained with 100 μg/ml of PI in the dark. Flow cytometry analyses were carried out on a FACSCalibur instrument using the ModFit program.

### Cell apoptosis analysis by annexin V/PI staining

Cell apoptosis was assessed by measuring membrane redistribution of phosphatidylserine using an Annexin V-FITC apoptosis detection kit according to the manufacturer’s protocol. In brief, cells (1 × 10^6^/well) were plated in six-well plates and allowed to attach overnight, Cells were treated with 0.1, 1 and 10 mg/ml Dasatinib for 24 h, washed twice with chilled PBS, resuspended in 250 μl of binding buffer, and stained with staining solution containing Annexin V-FITC and PI. After incubation in the dark for 30 min, cells were analyzed by FACSCalibur instrument.

### Hoechst 33258 staining in situ detection of cell injury

The human laryngeal cancer line Hep-2 was cultured to subconfluence in a six chamber slide with 10 % FCS containing DMEM. This was then added to (1) serum-free DMEM; (2) 0.1 mg/ml Dasatinib; (3) 1 mg/ml Dasatinib; and (4) 10 mg/ml Dasatinib. After treatment with Dasatinib or DMEM control, cells were fixed with cold methanol and acetic acid (3/1, v/v) at 4 °C overnight and stained with Hoechst 33258 for 30 min in the dark, washed again in PBS, and finally observed with a fluorescence microscope (Olympus, Japan).

### Western blot analysis

The human laryngeal cancer line Hep-2 was cultured to subconfluence in a 60 mm culture dish with 10 % FCS containing DMEM. The medium was then changed to either (1) serum-free DMEM; (2) 0.1 mg/ml Dasatinib; (3) 1 mg/ml Dasatinib; and (4)10 mg/ml Dasatinib. Protein was extracted in a standard lysis buffer with proteinase inhibitors (sodium orthovanadate, phenylmethylsulfonyl fluoride, leupeptin, and aprotinin obtained from BioShop Burlington, ON, Canada), 20 mg of protein lysate was electrophoresed with a 12 % SDS-PAGE gel, transferred to a nylon membrane, and probed with an antibody for total Src, p-Src, Bcl-2, Bax, caspase-3, and caspase-8. Following incubation with the secondary antibody, blots were developed by an ECL western blot substrate kit (Abcam, USA).

### Antitumor activity in the nude mouse tumor xenograft model

All animal studies were carried out in accordance with the “Guide for the Care and Use of Laboratory Animals.” Hep-2 cells, 5 × 10^6^ cells per 0.2 ml in PBS, were injected subcutaneously into the right armpit. Tumor size was measured every other day in two dimensions using caliper, and tumor volume was calculated using the formula, *ab* ^2^/2, where *b* is the smaller dimension. When tumors reached about 150 mm^3^, animals were randomized (five animals/group) into four groups. Rats in the normal saline (NS) group were injected with NS into the caudal vein for 22 days. Rats in the cisplatin group were treated with 100 mg/m^2^ of cisplatin injected into the caudal vein on the first day, followed by 21 days of NS administration. Rats in the Dasatinib group were injected with 10 mg/m^2^ of Dasatinib into the caudal vein for 22 days. Rats in the cisplatin + Dasatinib group were treated with 100 mg/m^2^ of cisplatin on the first day and 10 mg/m^2^of Dasatinib for 22 days injected into the caudal vein.

### Tumor inhibition rate

The inhibitory effect on tumor growth was evaluated by the tumor inhibition rate. Twenty-two days after administration, rats were anesthetized with ethyl ether and killed, and the whole body and tumor were weighed immediately. The inhibition rates of FAT solid tumor growth were calculated according to the formula, inhibition rate (%) = (1−mean weight of tumor in the drug treated groups/mean weight of tumor in the NS group) × 100 %.

### Statistical analysis

All values are expressed as mean ± SD. Statistical analysis was performed using the Student’s *t* test. A value of *p* < 0.05 was considered significant.

## Results

### Dasatinib inhibited cellular proliferation in a dose- and time-dependent manner

**Fig. 1:**
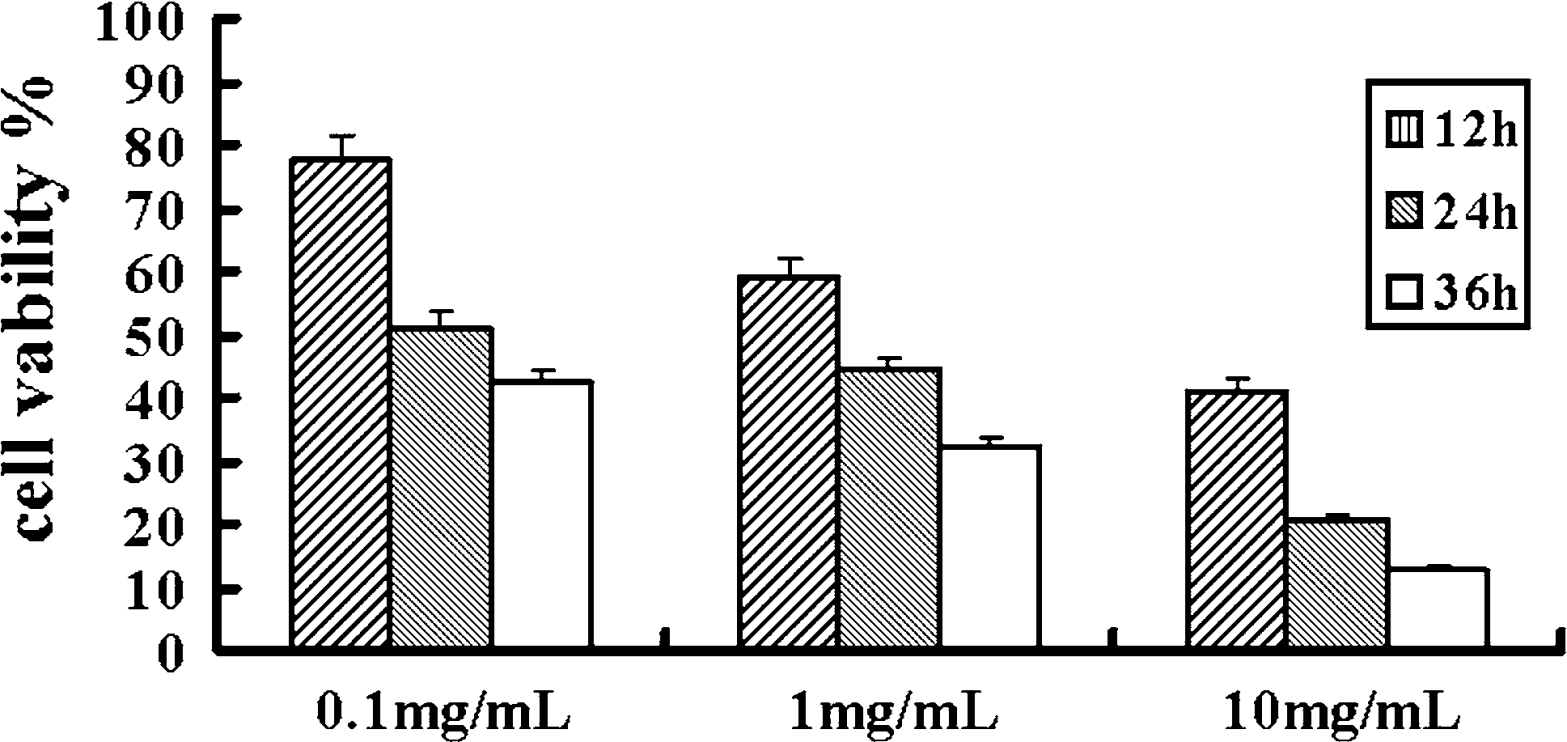
Viability of Hep-2 cells after treatment with Dasatinib. Hep-2 cells were treated with Dasatinib (0.1, 1.0, 10 mg/mL) for 12, 24 and 36 h. MTT assays were performed in triplicate for each data point

Accumulated evidences have shown that Dasatinib inhibits the growth of numerous human cancer cell lines [8]. To evaluate the effect of Dasatinib on cell growth of human laryngeal cancer cell line Hep-2, we employed MTT assay to assess cell growth inhibition. Hep-2 cells were grown in the presence or absence of various concentrations of Dasatinib for 1–2 days and showed dose -and time-dependent inhibition of cell proliferation (Fig. 1).

### Dasatinib-induced Hep-2 cells G0/G1 arrest

**Fig. 2:**
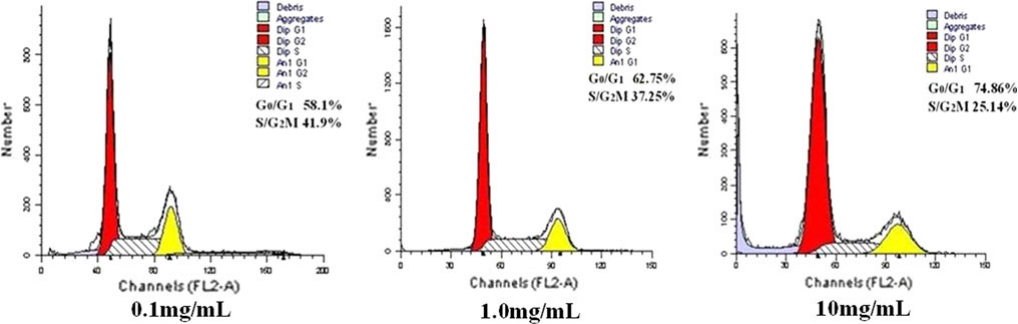
Effect of dasatinib on the cell cycle progression in Hep-2 cell line. Cells were blocked at the G1 phase transition by dasatinib. The cells were incubated for 24 h with dasatinib and, subsequently, the cell cycle profile was analyzed by flow cytometry. The percentages of cells in the G0/G1-phase and in the S-, G2- and M-phases are shown. Dasatinib induced a G1 cell cycle arrest in all of the cells tested

Most anticancer agents exhibit their inhibitory effects on tumor cell growth by inducing cell cycle arrest and apoptosis. A recent report has demonstrated that Dasatinib induces G0/G1 arrest and apoptosis in human breast cancer cell lines [9]. To gain insights into the mechanism by which cell reduction is achieved, we investigated the effect of Dasatinib on cell cycle distribution by FACS analysis, and the results were summarized in (Fig. 2). A 24 h exposure of Hep-2 cells to different concentrations of Dasatinib caused an enrichment of cells in G0/G1 phase in a dose-dependent manner (*P* < 0.05 vs control), accompanied by a reduction in S/G2 M phase cells (*P* < 0.05 vs. control).

### Annexin V/PI staining for cell apoptosis

**Fig. 3:**
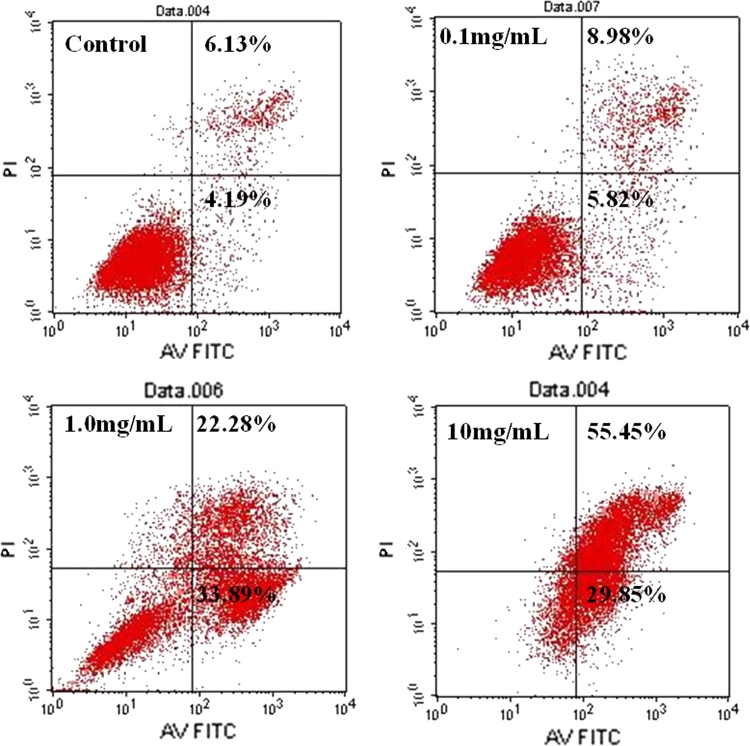
Induction of apoptosis in cells after Dasatinib treatment. Apoptosis of cells was evaluated by measuring the exposure of phosphatidylserine on the cell membranes using an Apoptosis Detection Kit. The apoptosis rates of the cell lines treated with 0.1, 1.0, 10 mg/mL Dasatinib increased remarkably

To demonstrate the cell apoptosis induced by Dasatinib, we employed flow cytometry analysis with Annexin V/PI staining. Following FACS, Xuorescence of PI was plotted over Annexin V-FITC Xuorescence (Fig. 3). Healthy cells have low FITC Xuorescence and low PI Xuorescence (Q3). Early apoptotic cells have high FITC Xuorescence but low PI Xuorescence (Q4). Late apoptotic cells have high FITC Xuorescence and high PI Xuorescence (Q2). Dead cells have low FITC Xuorescence but high PI Xuorescence (Q1). As shown in Fig. 3, the percentage of apoptotic cells (Q2 + Q4) induced by Dasatinib significantly increased with the increasing concentration (*P* < 0.05, or 0.01 vs. control).

### In situ detection of cell injury

**Fig. 4:**
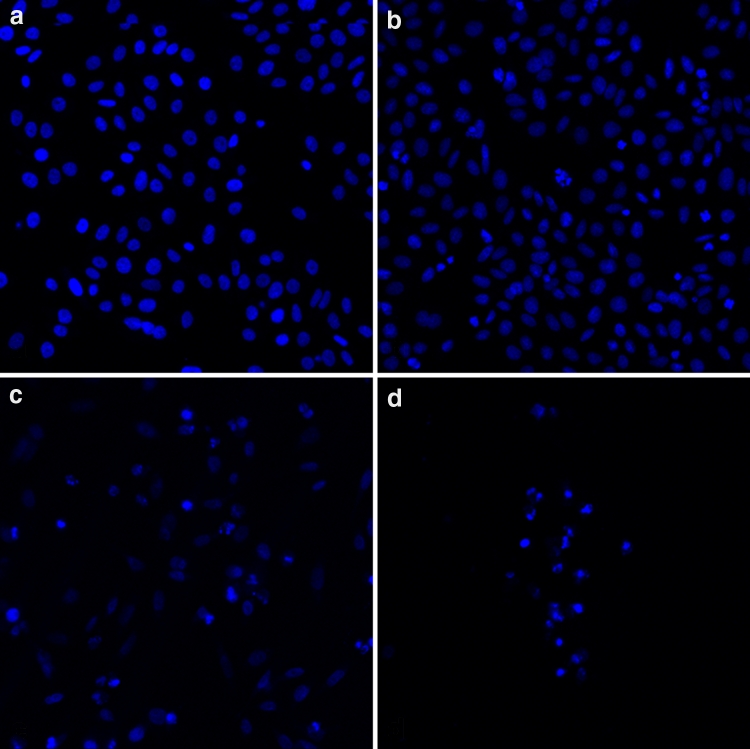
Cell apoptosis observed by Hoechst 33258 staining. Hep-2 cells were treated with solvent control **a**, 0.1 **b**, 1 **c**, and 10 mg/mL **d** Dasatinib for 24 h. From 0 to 10 mg/mL, the number of apoptotic cell increased gradually, while the number of total cells decreased

After Hep-2 cells were treated with Dasatinib (0.1, 1, 10 mg/ml) for 24 h, marked morphological changes associated with cell injury such as condensation of chromatin, nuclear fragmentation, and apoptotic bodies were clearly observed using Hoechst 33258 staining (Fig. 4). Compared with the control group, a significant increase in the number of impaired cells was noted following Dasatinib treatment.

### Dasatinib reduced the level of Src activation

**Fig. 5:**
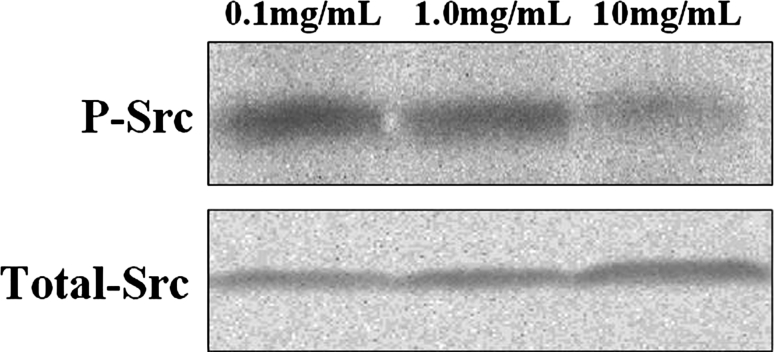
Effects of Dasatinib on P-Src and Total-Src protein expression in Hep-2 cells. Hep-2 cells were treated with Dasatinib (0.1, 1.0 and 10 mg/mL) for 24 h. The protein levels were monitored by western blot analysis

Given the Hep-2 cell line to concentration of Dasatinib, we compared the expression of total Src and P-Src level. We found that the Src activation (i.e., the level of P-Src), but not total Src expression, was correspondingly inhibited by Dasatinib in a dose-dependent manner (Fig. 5).

### Dasatinib modulates the expression of apoptosis-related proteins in Hep-2 cells

**Fig. 6:**
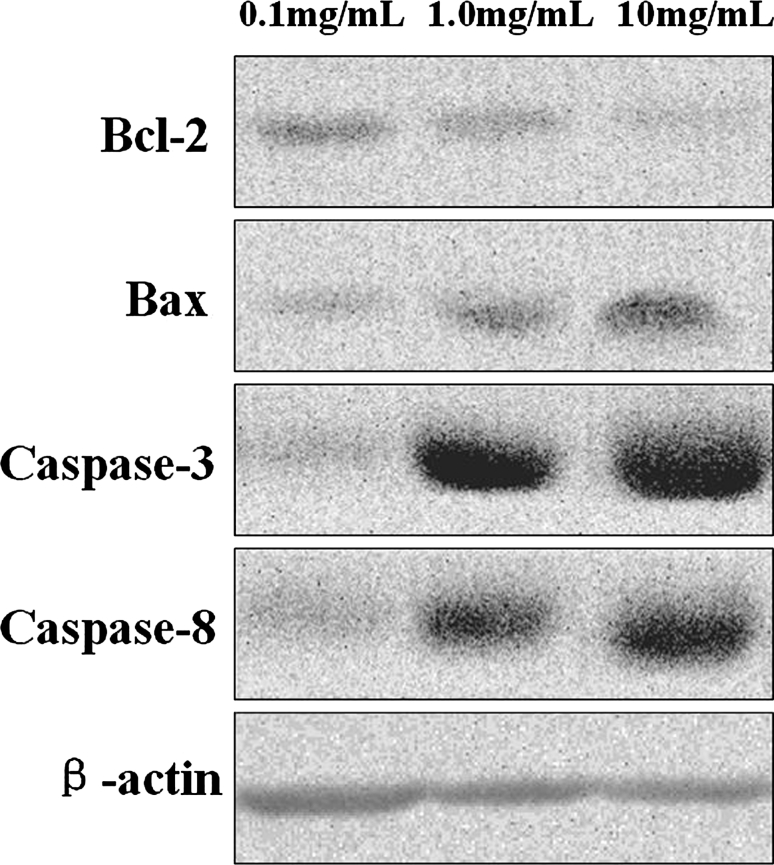
Western blot analysis of Bcl-2, Bax, Caspase-3, and Caspase-8 proteins. Hep-2 cells were treated with 0.1, 1, and 10 mg/mL Dasatinib for 24 h. The cells were harvested and processed for Western blotting

The mechanisms that underlie the combined effects of dasatinib on apoptosis-related proteins (caspase-3, caspase-8, Bax, bcl-2) were investigated. The levels of these proteins were evaluated using western blot analysis. Figure 6 shows that caspase-3, caspase-8, and Bax protein levels increased after 48 h of treatment with Dasatinib, while the level of bcl-2 protein decreased. Beta-actin was used as the loading control.

### Antitumor effect of Dasatinib on Hep-2 xenograft model

**Fig. 7:**
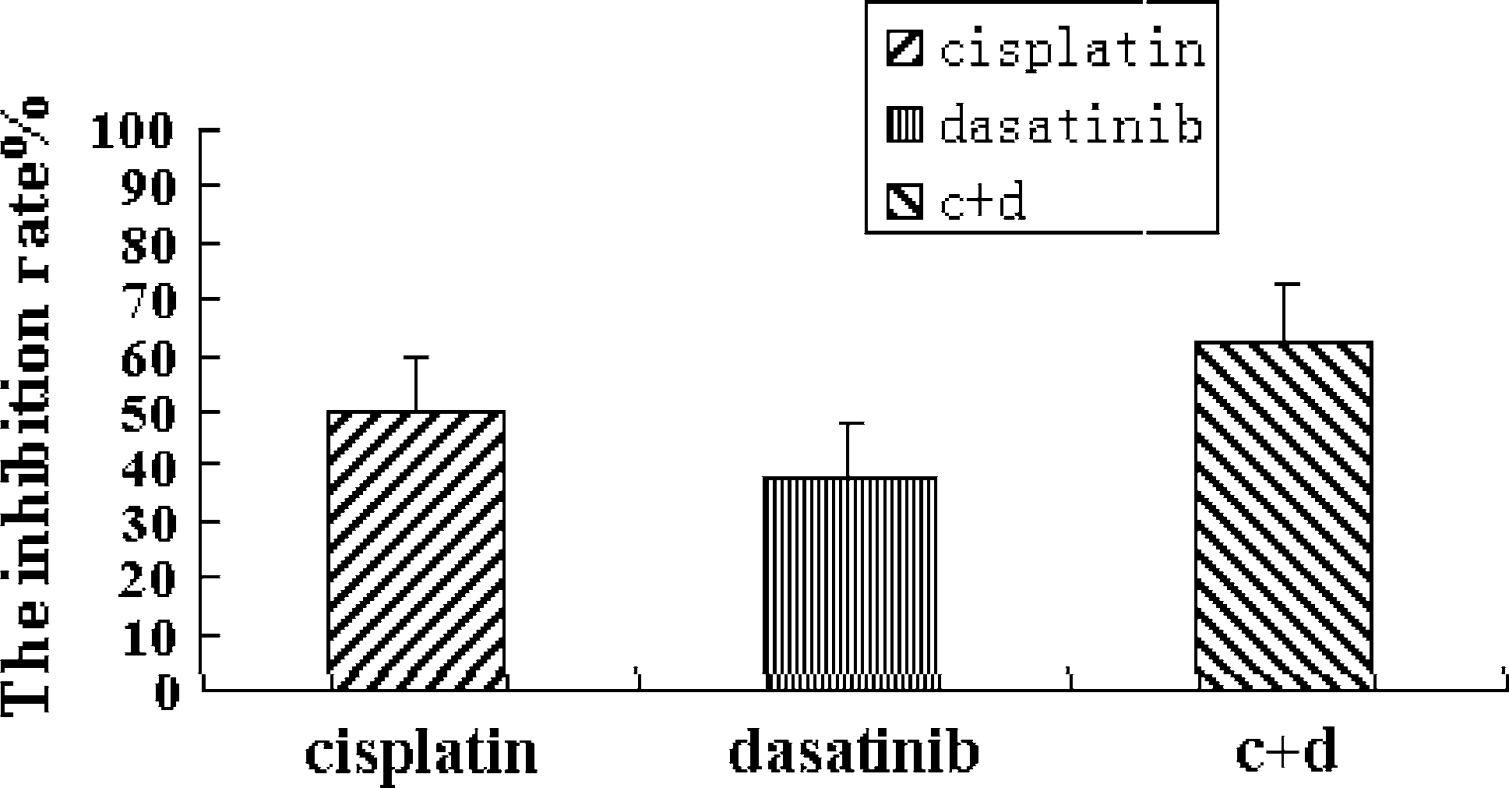
Antitumor effect of Dasatinib on Hep-2 xenograft model. Inhibition rate (%) = (1−mean weight of tumor in the drug treated groups/mean weight of tumor in the NS group) × 100 %

Twenty-two days after administration of Dasatinib, the mean tumor weights in the treatment groups decreased significantly compared with those in the control group (*p* < 0.05). In cisplatin group, the Dasatinib group, and the cisplatin + Dasatinib group, the tumor inhibition rates were 50, 37.5 and 62.5%, respectively. The difference in tumor inhibition was significant between the cisplatin group and the cisplatin + Dasatinib group (*p* < 0.05) (Fig. 7).

## Discussion

The non-receptor tyrosine kinase Src is known to promote cell proliferation, survival, and decreased adhesion, as well as increased motility, invasiveness, and angiogenesis, all of which promote the neoplastic phenotype [10]. Kinases are also excellent targets for anticancer therapy, as they work as a molecular switch, their regulation is reversible and rapid, and does not require new protein synthesis. Based on molecular targeting therapy, improper patient selection by predictive biomarkers leads to the success [11].

In the present study, we first examined the inhibitory effect of Dasatinib on cell viability and proliferation of Hep-2 cells. Using MTT assay, we observed that Dasatinib inhibited Hep-2 cells proliferation in a dose- and time-dependent fashion (Fig. 1). To our knowledge, this is the first study to demonstrate that Dasatinib exhibits antitumor effect on human laryngeal cancer cell line. A recent report has demonstrated that Dasatinib induces G0/G1 arrest and apoptosis in human breast cancer cell lines [12]. To determine whether Dasatinib induces cell cycle arrest and apoptosis in Hep-2 cell line, we performed a series of experiments on cell cycle distribution and apoptosis. Flow cytometry analysis showed that the administration of Dasatinib resulted in an accumulation of cells at G0/G1 phase (Fig. 2). Fluorescent microscopy observation by Hoechst 33258 staining showed that treatment of Hep-2 cells with Dasatinib led to the occurrence of typical morphological changes of apoptosis (Fig. 4). Flow cytometry analysis with Annexin V/PI staining indicated that Dasatinib-induced Hep-2 cell apoptosis in a dose- and time-dependent manner (Fig. 3). These data suggested that the inhibitory of Dasatinib against this cell line was due to the induction of cell cycle arrest as well as apoptosis.

To better understand the signaling mechanism that affected Dasatinib, we examined the effects of agent on the phosphorylation status of Src. As expected, Dasatinib treatment of Hep-2 cells inhibited serum-induced Src phosphorylation. We found that the Src activation (i.e., the level of P-Src), but not total Src expression, was correspondingly inhibited by Dasatinib in a dose-dependent manner (Fig. 5). These suggested degradation of P-Src mediated Dasatinib-induced apoptosis in Hep-2 cells. Dasatinib inhibited Hep-2 cells proliferation, induced G0/G1 arrest, and apoptosis by reducing the level of Src activation.

Dasatinib may induce apoptosis through mitochondria-dependent and death receptor-dependent apoptotic pathways. Dasatinib suppresses cancer cell growth by inhibiting proliferation through the promotion of caspase-dependent apoptosis. Caspases are cytoplasmic aspartate-specific cysteine proteases, and it plays an important role in apoptosis [13]. The death receptor-dependent apoptotic pathway is triggered at the cell surface and requires activation of caspase-8, whereas the mitochondrion-dependent pathway is initiated by the release of mitochondrial cytochrome *c* into the cytoplasm and requires activation of caspase-9. Subsequently, caspase-8 or -9 can activate caspase-3, which in turn targets and degrades specific and vital cellular proteins, ultimately resulting in nuclear DNA degradation and apoptotic cell death [14]. It is obvious that activation of caspases is central to the execution of apoptosis [15]. Bcl-2, an inhibitor of the mitochondrial apoptosis pathway, exerts its action by blocking proapoptotic counterparts, which in turn prevents the release of cytochrome c and the activation of caspases [16]. Bax is a death promoter, which is neutralized by heterodimerization with Bcl-2. Bax translocates into the outer mitochondrial membrane followed by leakage of cytochrome *c* from the mitochondria into the cytosol [17]. Caspase-9 and caspase-3 are activated sequentially, and this event then leads to the breakdown of chromosomal DNA. There is a significant possibility that Dasatinib-mediated anti-apoptosis of Hep-2 cells is the result of regulation of Bcl-2 and Bax. Hence, identification of the target compounds is necessary. We found a negative correlation between Bcl-2 expression and Dasatinib-induced apoptosis.

In the evaluation of the efficacy of Dasatinib in vivo, drug treatment in the nude mouse model utilizing Hep-2 cells produced a significant reduction in tumor burden. The Dasatinib-induced reduction in tumor weight was comparable to that produced by the conventional chemotherapeutic agent DDP. In this study, DDP and Dasatinib were combined to enhance antitumor activities. This new prescription (DDP + Dasatinib) was better than DDP alone in terms of therapeutic efficacy. Complexities of cell signaling in advanced cancer including recurrent HNSCC may result in the activation of feedback mechanisms. Therefore, the combination of molecular targeted therapy is a rational approach. We found that Dasatinib can enhance the effect of Cisplatin providing the basis for combination of Dasatinib with Cisplatin to treat HNSCC. A pharmacodynamic study shows that Src activity is inhibited by Dasatinib (at a dosage of 100 mg/day) rapidly, but recovered within 6–12 h [18]. Because Dasatinib 100 mg/day is well-tolerated, dose escalation is a possible strategy to overcome the recovery of Src activity.

The antitumor activity against Hep-2 cells mediated via Dasatinib was due to the induction of cell cycle arrest as well as apoptosis. The possible mechanisms underlying the action might be attributed to the suppression of Src phosphorylation relevant to the regulation of cell cycle and apoptosis. Dasatinib + DDP are more effective in inhibiting the growth of Hep-2 cells than either agent alone.
